# The genus *Keilbachia* Mohrig from Mainland China, with descriptions of two new species (Diptera, Sciaridae)

**DOI:** 10.3897/zookeys.52.362

**Published:** 2010-07-30

**Authors:** Su-Jiong Zhang, Junhao Huang, Hong Wu, Yi-ping Wang

**Affiliations:** College of Forestry and Biotechnology, Zhejiang A & F University, Hangzhou 311300, China; College of Forestry and Biotechnology, Zhejiang A & F University, Hangzhou 311300, China; College of Forestry and Biotechnology, Zhejiang A & F University, Hangzhou 311300, China; College of Forestry and Biotechnology, Zhejiang A & F University, Hangzhou 311300, China

**Keywords:** Diptera, Sciaridae, Keilbachia, new species, Mainland China, Oriental Region

## Abstract

Seven species of Keilbachia Mohrig are recognized, and among them, two new species, Keilbachia subacumina Wu & Zhang, **sp. n.** and Keilbachia fengyangensis Wu & Zhang, **sp. n.** are described and illustrated. Five species, Keilbachia orthonema, Keilbachia flagrispina, Keilbachia demssia, Keilbachia oligonema and Keilbachia acumina are reported for the first time from China. A key to the 15 Chinese species of this genus is also provided.

## Introduction

The genus Keilbachia was firstly proposed for Keilbachia nepalensis Mohrig from Nepal (Mohrig and Martens 1987: 483). Subsequently, additional species are described from the Oriental, Palaearctic, Neotropical, and Australian Regions. Menzel and Martens (1995) described two species from Nepal-Himalaya. Mohrig et al. (1999) described four species from Nepal. Menzel and Mohrig (2000) transferred two Palaearctic species to Keilbachia. Mohrig (2004) described one new species from Papua New Guinea. Mohrig et al. (2004) described one new species from Dominica. Vilkamaa et al. (2006) described eight new species from Myanmar. Hippa and Vilkamaa (2007a, b) described 15 species from Oriental regions and reassigned one Nearctic species to the genus. Rudzinski (2008) described five new species from Taiwan. Vilkamaa et al. (2009) reviewed the genus, adding 11 new species from Oriental Region and one more species by combination. Therefore, 52 species of the genus have been recorded until now, mostly from the Oriental Region, including eight species that occur in Taiwan.

In this study, seven species of Keilbachia are recognized from Mainland China. Among them, two new species, Keilbachia fengyangensis and Keilbachia subacumina are described and illustrated. Five species, Keilbachia flagrispina, Keilbachia demssia, Keilbachia orthonema, Keilbachia oligonema and Keilbachia acumina are reported for the first time from China. An additional 8 species of Keilbachia are known from Taiwan China: Keilbachia adjuncta Vilkamaa, Menzel & Hippa (2009: 5); Keilbachia ferrata (Hippa & Vilkamaa 1994: 50, Camptochaeta); Keilbachia grandiosa Rudzinski (2008: 347); Keilbachia praedicata Rudzinski (2008: 348); Keilbachia profana Rudzinski (2008: 349); Keilbachia sasakwawai (Mohrig & Menzel 1992: 21, Corynoptera); Keilbachia subferrata Rudzinski (2008: 346) and Keilbachia ulcerate Rudzinski (2008: 349).

## Materials and methods

All specimens were collected by sweeping in the field and preserved in 75% ethanol. They were mounted on glass slides in xylol-based Canada balsam after clearing in creosote. The heads of specimens from Yunnan province were bleached in 10% NaOH for about 24 hours at room temperature. The specimens were observed and measured under a Nikon SMZ1500 stereoscopic microscope. The illustrations were prepared under a Nikon Eclipse 50i optical microscope, with an attached drawing tube. The terminology follows Hippa and Vilkamaa (2007b). The length of 4th flagellomere is taken from the apex of the neck to the base of the body. The wing length is the straight distance from the humeral angle to the apical angle. The body length is the straight distance between apex of head and apex of hypopygium. The type specimens designated in the present study are deposited in the collection of the Laboratory of Forest Protection, Zhejiang A & F University, Hangzhou, Zhejiang province, China [ZAFU].

## Results and discussion

### Key to Chinese Species of Keilbachia (Based on Males)

**Table:** 

1.	Gonostylus with subapical megasetae	3
–	Gonostylus with no subapical megasetae	2
2.	Gonostylus with one mesial megaseta	Keilbachia praedicata
–	Gonostylus with two mesial megasetae	Keilbachia oligonema
3.	Gonostylus with one or two subapical megasetae	5
–	Gonostylus with three or more subapical megasetae	4
4.	Gonostylus with one mesial megaseta on middle and another one at the base of gonostylus	Keilbachia profana
–	Gonostylus with only one mesial megaseta at the base of gonostylus	Keilbachia ulcerate
5.	Gonostylus with two subapical megasetae	Keilbachia adjuncta
–	Gonostylus with two subapical megasetae	6
6.	Mesial megaseta of gonostylus short, shorter than maximal width of gonostylus	Keilbachia orthonema
–	Mesial megaseta of gonostylus long, at least as long as maximal width of gonostylus	7
7.	Subapical megasetae of gonostylus close to each other, both at apical fourth of gonostylus	11
–	Subapical megasetae of gonostylus widely apart, basal most one at apical third or apical half of gonostylus	8
8.	Tegmen modified, slightly broader subbasally than subapically	Keilbachia sasakawai
–	Tegmen simple, much broader subbasally than subapically	9
9.	Basal body of mesial megaseta of gonostylus long and slender	Keilbachia subferrata
–	Basal body of mesial megaseta of gonostylus short and stout	10
10.	Basalmost subapical megasetae at apical half of gonostylus (Fig. 5)	Keilbachia subacumina
–	Basalmost subapical megasetae at apical third of gonostylus (Fig. 6)	Keilbachia acumina
11.	Subapical megasetae of gonostylus subequal in size	13
–	Subapical megasetae of gonostylus not equal in size	12
12.	Apicalmost subapical megaseta of gonostylus slender	Keilbachia ferrata
–	Apicalmost subapical megaseta of gonostylus stout	Keilbachia grandiosa
13.	Apex of gonostylus rounded and broad	Keilbachia flagrispina
–	Apex of gonostylus pointed and	14
14.	Mesial megaseta of gonostylus long and strongly curved (Fig. 10)	Keilbachia fengyangensis
–	Mesial megaseta short and slightly curved	Keilbachia demissa

#### Keilbachia flagrispina

Keilbachia flagrispina Mohrig, in Mohrig, Röschmann & Rulik 1999: 198.

##### Diagnostic characters.

(Male). Body length 1.64–1.71mm; wing length 1.36–1.41mm.

Eye bridge 3–4 facets wide. Length/width of 4th flagellomere 2.19–2.32.

Anterior pronotum with 5–6 setae. Episternum 1 with 3–4 setae.

c/w 0.69–0.72, R1/R 0.71–0.76, r-m with one seta.

The mesial megaseta on gonostylus very long and curved, nearly three times as long as the width of gonostylus. The basal body of mesial megaseta is not distinct. Tegmen simple, much broader subbasally than subapically.

##### Specimens examined.

China, Yunnan, Baoshan, Mts. Gaoligongshan, 24°49.729'N; 98°46.074'E, sweep-net 11.V.2009. 4 males, Man-Man Wang [SM00878–00880, SM00882] (ZAFU); 3 males, Su-Jiong, Zhang [SM00886, SM00902–00903] (ZAFU).

##### Distribution.

China (Yunnan), Myammar, Nepal.

##### Biology.

Unknown.

##### Remarks.

This species is new to China, which was firstly described from Nepal, based on two males. It is similar to Keilbachia ferrata (Hippa & Vilkamaa, 1994) in having two subapical megaseta and a long mesial megaseta, but Keilbachia flagrispina can be separated by the mesial megaseta very long and strongly curved, and two subapical megaseta subequal in length on gonostylus. The materials examined from China do not show distinct variation, but we found the Chinese specimens are much smaller in body length, which is 1.64–1.71 mm, while 2.5 mm in Nepal materials.

#### Keilbachia demissa

Vilkamaa, Komarova & Hippa

Keilbachia demissaVilkamaa, Komarova & Hippa, 2006: 45.

##### Diagnostic characters.

(Male). Body length 1.73–1.78 mm; wing length 1.47–1.49 mm.

Eye bridge 3–4 facets wide. Prefrons with 10–12 setae. Length/width of 4th flagellomere 2.17–2.41.

Anterior pronotum with 5–6 setae. Episternum 1 with 7–8 setae.

Length of spur/width of fore tibia 1.20–1.27. Length of metatibia /length of thorax 1.05–1.12.

c/w 0.62–0.64, R1/R 0.52–0.56, r-m with no setae.

The mesial megaseta on gonostylus long and slightly curved, basal body long. Tegmen simple, much broader subbasally than subapically.

##### Specimens examined.

China, Yunnan, Baoshan, Mts. Gaoligongshan, 24°49.729'N; 98°46.074'E, sweep-net, 11.V.2009. 6 males, Su-Jiong Zhang [SM00856, SM00859, SM00862, SM00869, SM00875, SM00881] (ZAFU); 1 male, Man-Man Wang [SM00888] (ZAFU).

##### Distribution.

China (Yunnan), Burma.

##### Biology.

Unknown.

##### Remarks.

This species is new to China, which was firstly described from Burma based on seven males. It is similar to Keilbachia scutica Vilkamaa, Komarova & Hippa, 2006 and Keilbachia flagrispina by the tegmen broadest subbasally (Mohrig et al. 1999; Vilkamaa, Komarova and Hippa 2006). But it differs in having the mesial megaseta of the gonostylus much shorter and less strongly curved. The materials examined in China do not show distinct intraspecies variation, but the apical of gonostylus in the specimens SM00881 and SM00856 is more attenuated and curved than the other specimens.

#### Keilbachia orthonema

Hippa & Vilkamaa

Keilbachia orthonemaHippa & Vilkamaa, 2007b: 66.

##### Diagnostic characters.

(Male). Body length 1.76–1.82 mm; wing length 1.35–1.37 mm.

Eye bridge 3–4 facets wide. Prefrons with 9–11 setae. Length/width of 4th flagellomere 2.47–2.53.

Anterior pronotum with 4–5 setae. Episternum 1 with 5–6 setae.

Length of spur/width of protibia 1.76–1.81. c/w 0.79–0.82, R1/R 0.71–0.73, r-m with 0–1 seta.

The mesial megaseta on gonostylus short and straight, slightly longer than its basal body. Two slender megasetae at apical forth of gonostylus. Tegmen slightly broader subbasally than subapically.

##### Specimens examined.

China, Yunnan, Yingjiang, Tongbiguan, 24°36.004'N; 97°39.139'E, sweep-net, 20.V.2009. 6 males, Su-Jiong Zhang [SM00657–00658, SM00663–00664, SM00670, SM00680] (ZAFU); 3 males, Man-Man Wang [SM00653, SM00666–00667] (ZAFU).

##### Distribution.

China (Yunnan), Malaysia.

##### Biology.

Unknown.

##### Remarks.

This species is new to China, which was firstly described from Sabah, Malaysia, based on two male specimens. It is similar to Keilbachia apprima Vilkamaa, Komarova & Hippa, 2006 from Vietnam by sharing a short mesial megaseta (Hippa and Vilkamaa 2007b), but Keilbachia orthonema can be distinguished by mesial megaseta longer and much less curved, and apical forth of gonostulus with two slender megasetae. The Chinese material examined does not show distinct intraspecies variation, but the two megasetae at the apical forth of the gonostylus are stronger than in Malaysia materials, judging from the figures prepared by Hippa and Vilkamaa (2007b). What’s more, length/width of 4th flagellomere is 2.47–2.53, smaller than in Malaysia materials, which is about 3 times as long as wide.

#### Keilbachia acumina

Vilkamaa, Menzel & Hippa

Keilbachia acuminaVilkamaa, Menzel & Hippa, 2009: 4.

##### Diagnostic characters.

(Male). Body length 1.51–1.57 mm; wing length 1.25–1.28 mm.

Eye bridge three facets wide. Prefrons with 3–5 setae. Length/width of 4th flagellomere 2.35–2.71.

Anterior pronotum with 3–4 setae. Episternum 1 with 4–5 setae.

Length of spur/width of protibia 1.55–1.57.

c/w 0.64–0.65, R1/R 0.72–0.75, r-m with no setae.

Gonostylus with two megasetae widely apart, one at subapical and stout, the other at apical third and slender (Fig. 6). Basal third of gonostylus excavated, with a long and strongly curved subbasal mesial megaseta on broad basal body. Tegmen simple, much broader subbasally than subapically, with sparsely placed teeth.

##### Specimens examined.

1 male, China, Zhejiang, Linan, Mt. Xijingshan, 30°23'N; 119°72'E, sweep-net, 21.VI.2008, Su-Jiong Zhang [SM00018] (ZAFU); 1 male, China, Zhejiang, Lishui, Mt. Jiulongshan, 28°59'N; 119°25'E, sweep-net, 10.X.2008, Su-Jiong Zhang [SM00114] (ZAFU); 1 male, Yunnan, Tengchong, Shaba, Mt. Tiantaishan, 25°24.524'N; 98°42.735'E, sweep-net, 13.V.2009, Su-Jiong Zhang [SM00933] (ZAFU).

##### Distribution.

China (Zhejiang, Yunnan), Japan.

##### Biology.

Unknown.

##### Remarks.

The species is similar to Keilbachia subferrata Rudzinski and Keilbachia ferrata (Hippa & Vilkamaa) by having a rather long subbasal mesial megaseta on a large basal body, But it can be distinguished from Keilbachia subferrata by the smaller mesial megaseta in a more apical position (Vilkamaa et al. 2009), and differs from Keilbachia ferrata by having the basal body slightly smaller, and the socket of the apical megaseta more distinct (Vilkamaa et al. 2009). Vilkamma et al. (2009) mentioned Keilbachia acumina shows intraspecies variation in the structure of the gonostylus and the length of the flagellomeres. The variations are also examined in the Chinese materials, that the position of basalmost subapical megasetae varies in the apical third of gonostylus and the length of the 4th flagellomere among 67.63–87.58 um.

#### Keilbachia subacumina

Wu & Zhang sp. n.

urn:lsid:zoobank.org:act:878985DF-CC83-4052-9486-06582BA13734

Figs 1–5 

##### Description.

###### Male.

Body length 1.81–2.32 mm; wing length 1.49–1.52 mm.

###### Color.

Head, thorax and abdomen brown; antenna, palpus, coxae, and hypopygium yellowish-brown; leg yellow; wing fumose.

###### Head (Figs 1, 2).

Eye bridge 3–4 facets wide. Prefrons with 5–6 setae, clypeus with 0–1 seta. Palpus three-segmented. Basal segment with one seta, with a narrow sensory pit, 2nd segment with 4–6 setae, 3rd segment with 6–7 setae. Length/width of 4th flagellomere 2.74–2.79.

###### Thorax.

Anterior pronotum with 5–6 setae. Episternum 1 with 6–7 setae.

###### Legs.

Apex of protibia (Fig. 3). Length of spur/width of protibia 1.79–1.83. Length of profemur/length of protibia 0.76–0.79. Length of metatibia /length of thorax 1.23–1.31.

###### Wings.

Width/length 0.44–0.49. c/w 0.71–0.77, R1/R 0.96–0.98. r-m with 1–2 setae.

###### Abdomen.

Sternite 8 with 10–11 setae. Gonostylus and gonocoxa subequal in length. Gonostylus with two megasetae widely apart, one at apex and stout, the other at apical half of gonostylus and slender. Basal third of gonostylus excavated, with a long and strongly curved mesial megaseta on broad basal body. Tegmen simple, much broader subbasally than subapically, with sparsely placed teeth. (Figs 4, 5).

**Figures 1–6.**
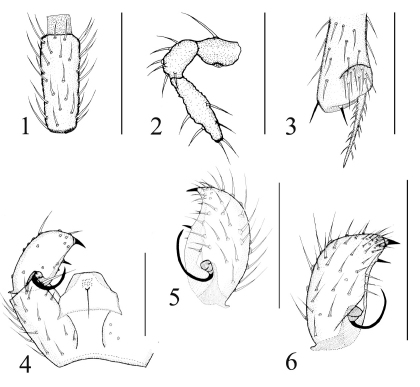
**1–5**, Keilbachia subacumina, male. **1** 4th flagellomere, lateral view **2** palpus, lateral view **3** apex of protibia, prolateral view **4** part of hypopygium, ventral view **5** gonostylus, ventral view. **6** Keilbachia acumina, male, gonostylus, ventral view. Scale bar = 0.1 mm.

##### Specimens examined.

Holotype, male. China, Zhejiang, Linan, Mt. Xijingshan, 30°23'N; 119°72'E, sweep-net, 21.VI.2008, Su-Jiong Zhang [SM00025] (ZAFU). Paratypes. 2 males, same data as holotype [SM00018, SM00024] (ZAFU); 5 males, same data as holotype but 19.VII.2008 [SM00057–00061] (ZAFU). China, Zhejiang, Lishui, Mt. Fengyangshan, 28°04'N; 119°08'E. sweep-net, 3 males, 11.VIII.2008, Sheng-Long Liu [SM00282] (ZAFU); 1 male, 24.VIII.2008, Sheng-Long Liu [SM00231–00233] (ZAFU); 1 male, 01.VIII.2008, Xiao-Ling Niu [SM00306] (ZAFU). 1 male, China, Zhejiang, Lishui, Mt. Jiulongshan, 28°59'N; 119°25'E, sweep-net, 10.X.2008, Su-Jiong Zhang, [SM00114] (ZAFU).

##### Biology.

Unknown.

##### Remarks.

This species is very similar to Keilbachia acumina in the structure of the hypopygium (Fig. 5, 6), but Keilbachia subacumina can be distinguished by having the apex of gonostylus broader, and the stouter and shorter basalmost megaseta at the apical half of the gonostylus. What’s more, the anterior pronotum bears 5–6 setaein Keilbachia subacumina while 3–4 setae in Keilbachia acumina, and the r-m nervation of the wing with 1–2 setae in Keilbachia subacumina while bare in Keilbachia acumina. The structure of the hypopygium in the new species does not show distinct intraspecies variation. The species is named after its similarity to Keilbachia acumina. This epithet is an adjective.

#### Keilbachia fengyangensis

Wu & Zhang sp. n.

urn:lsid:zoobank.org:act:3BA818B3-5B93-495A-9117-BC26DDE09CF8

Figs 7–11 

##### Description.

###### Male.

 Body length 2.31–2.48 mm; wing length 1.92–1.94 mm.

###### Color.

 Head, thorax and abdomen brown; antenna, palpus, coxae, legs and hypopygium yellowish-brown; wing fumose.

###### Head (Fig. 7, 8).

Eye bridge 3–4 facets wide. Prefrons with 7–8 setae, clypeus with no setae. Palpus three-segmented. Basal segment with one seta, with wide sensory pit, 2nd segment with 4–6 setae, 3rd segment with 7–8 setae. Length/width of 4th flagellomere 2.91–2.94.

###### Thorax.

 Anterior pronotum with 4 setae, episternum 1 with 5–6 setae.

###### Legs.

 Apex of protibia (Fig. 9). Length of spur/width of protibia 1.45–1.49. Length of profemur/length of protibia 0.65–0.68. Length of metatibia/length of thorax 1.03–1.11.

###### Wings.

 Width/length 0.45–0.47. c/w 0.62–0.69; R1/R 0.83–0.91. r-m with one seta.

###### Abdomen.

 Sternite 8 with nine setae. Gonostylus longer than gonocoxa, slightly curved, with two slender apical and subapical megaseta. The basal third of gonostylus with a long and curved mesial megaseta on a narrow and short basal body. Tegmen higher than broad, with sparse placed teeth (Fig. 10, 11).

**Figures 7–11.**
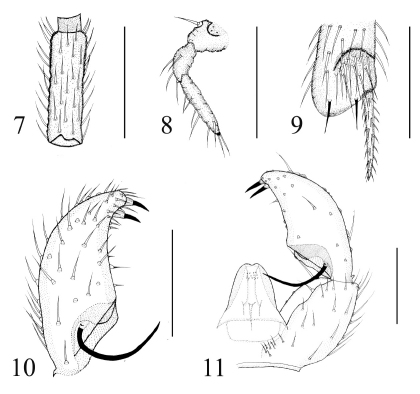
Keilbachia fengyangensis, male. **7** 4th flagellomere, lateral view **8** palpus, lateral view **9** apex of protibia, prolateral view **10** gonostylus, ventral view **11** part of hypopygium, ventral view. Scale bar = 0.1 mm.

##### Specimens examined.

Holotype, male. China, Zhejiang, Lishui, Mt. Fengyangshan, 28°04'N; 119°08'E, sweep-net, 26.IV.2008, Sheng-Long Liu [SM00342] (ZAFU). Paratypes. 2 males, same data as holotype [SM00335, SM00338] (ZAFU); 1 male, same data as holotype but 19.IV.2008 [SM00346] (ZAFU).

##### Biology.

Unknown.

##### Remarks.

This species is found only from Mt. Fengyangshan, Zhejiang. It is similar to Keilbachia demissa and Keilbachia flagrispina by having a long mesial megaseta, but it can be distinguished from Keilbachia demissa by having the megaseta of the gonostylus longer and more curved, and the tegmen with no distinct basolateral. The new species can be distinguished from Keilbachia flagrispina by the apex of gonostylus distinct attenuated, and the mesial megaseta shorter and not strongly curved. The species is named after its type locality (Mt. Fengyangshan).

#### Keilbachia oligonema

Hippa & Vilkamaa

Keilbachia oligonemaHippa & Vilkamaa, 2007a: 45.

##### Diagnostic characters.

(Male). Body length 1.71 mm; wing length 1.66 mm.

Eye bridge four facets wide. Prefrons with nine setae. Length/width of 4th flagellomere 3.30.

Anterior pronotum with five setae, episternum 1 with three setae.

Apex of protibia. Length of spur/width of protibia 1.48.

Width/length 0.40, c/w 0.73, R1/R 0.87. r-m with no setae.

Gonocoxa ventrally with a slight indication of an intercoxal lobe. Gonostylus nearly as long as gonocoxa, with no apical and subapical megaseta. The basal third of gonostylus excavated, with two long and curved mesial megaseta on a broad basal body. Tegmen simple, much broader subbasally than subapically.

##### Specimens examined.

1 male. China, Yunnan, Tengchong, Dahaoping, 24°55'N; 98°45'E, sweep-net, 22.V.2009, Man-Man Wang, [SM00757] (ZAFU).

##### Distribution.

China (Yunnan), Burma.

##### Biology.

Unknown.

##### Remarks.

The gonostylus of Keilbachia oligonema Hippa & Vilkamaa, 2007 with two long and curved mesial megaseta, and without apical and subapical megaseta. It is different from all the other species in the group of flagria, which has more than one mesial megaseta on the gonostylus. Keilbachia oligonema is unique in having different characters in width of the eye bridge, setosity of sternite 8, and the ventral intercoxal area of the hypopygium between the holotype and two additional specimens, from which the authors suspected they may represent two different species (Hippa and Vilkamaa 2007a). The same as the holotype of Keilbachia oligonema, the Chinese material has gonocoxa ventrally with a slight indication of an intercoxal lobe, but its four facets wide eye bridge, and six setose sternite 8 are similar to the additional materials.

## Supplementary Material

XML Treatment for Keilbachia flagrispina

XML Treatment for Keilbachia demissa

XML Treatment for Keilbachia orthonema

XML Treatment for Keilbachia acumina

XML Treatment for Keilbachia subacumina

XML Treatment for Keilbachia fengyangensis

XML Treatment for Keilbachia oligonema
